# Supplementary material for: CFAP45 deficiency causes situs abnormalities and asthenospermia by disrupting an axonemal adenine nucleotide homeostasis module
Source: Nat Commun. 2020 Nov 2;11:5520. doi: 10.1038/s41467-020-19113-0 (PMC7606486; doi:10.1038/s41467-020-19113-0)
Supplement: Supplementary file 1 — Supplementary Information [file 41467_2020_19113_MOESM1_ESM.pdf]

Supplementary Information

CFAP45 deficiency causes situs abnormalities and asthenospermia by disrupting an axonemal adenine nucleotide homeostasis module

Dougherty et al.

Individual	HGNC	RefSeq	Consequence	cDNA	Protein	Variant
OP-28 II1	*CFAP45*	NM_012337.2	nonsense (LOF)	c.721C>T	p.Gln241*	n.a.
OP-28 II1	*CFAP45*	NM_012337.2	nonsense (LOF)	c.907C>T	p.Arg303*	rs201144590
OP-985 II1	*CFAP45*	NM_012337.2	frameshift (LOF)	c.452_464delAGAAGGAGATGGT	p.Gln151Argfs*40	n.a.
TB-19 II1	*CFAP45*	NM_012337.2	frameshift (LOF)	c.1472_1477delAGAACCinsT	p.Gln491Leufs*5	n.a.
OI-81 II1	*CFAP52*	NM_145054.4	deletion (LOF)	c.70+1535_270+360del	p.His25Argfs*8	n.a.
OI-140 II1	*CFAP52*	NM_145054.4	deletion (LOF)	c.70+1535_270+360del	p.His25Argfs*8	n.a.
OI-142 II1	*CFAP52*	NM_145054.4	frameshift (LOF)	c.1303_1304del(G)2insG	p.Gly435Alafs*7	rs1360832162
OI-161 II1	*CFAP52*	NM_145054.4	missense	c.811G>A	p.Gly271Arg	rs140921334

Supplementary Table 1 | Genetic overview of motile ciliopathy individuals in this study. A 772-gene "ciliaproteome" NGS panel identifies compound heterozygous nonsense mutations (c.721C>T, p.Gln241*; c.907C>T, p.Arg303*) in *cilia and flagella associated protein 45* (*CFAP45*) in individual OP-28 II1 (**Fig. 1**), following both copy number and single nucleotide variant analysis. WES identifies homozygous frameshift *CFAP45* mutations in unrelated individuals OP-985 II1 (**Fig. 1**) and TB-19 II1 (**Supplementary Fig.1**). WES also identifies mutations in *cilia and flagella associated protein 52* (*CFAP52)* in four unrelated individuals in the motile ciliopathy cohort (**Supplementary Fig. 5**). These mutations are either ultra-rare or absent from the gnomAD or 1000 Genomes databases (indicated by n.a., not applicable) or present only in heterozygosity in few healthy controls. Respective minor allele frequency (MAF) for variants rs201144590, rs1360832162, and rs140921334 are 0.0001404, 0.000004, and 0.00003305. LOF indicates loss-of-function. *CFAP52* homozygous missense variant c.811G>A, pGly271Arg (rs140921334) is predicted to be deleterious or probably damaging by Provean and Polyphen webtool analysis, respectively; this variant is likely pathogenic (see **Supplementary Fig. 8i**). See also **Methods** for description of motile ciliopathy cohort, genetic analysis and prioritization of pathogenic variants.

Individual	Age	Gender	Mutation	Consanguinity	Otitis Media	Chronic Sinusitis	Chronic Cough	nNO
OP-28 II1	31	male	*CFAP45*	no	yes	yes	no	173.6
OP-985 II1	19	male	*CFAP45*	yes	no	yes	no	n.r.
TB-19 II1	19	male	*CFAP45*	yes	yes	n.r.	no	337.2
OI-81 II1	51	male	*CFAP52*	yes	n.r.	n.r.	yes	491.4
OI-140 II1	18	female	*CFAP52*	yes	n.r.	yes	n.r.	90
OI-142 II1	15	male	*CFAP52*	yes	yes	yes	yes	210
OI-161 II1	9	male	*CFAP52*	yes	yes	yes	n.r.	36

Supplementary Table 2 | Clinical overview of motile ciliopathy individuals in this study. Individuals with *CFAP45* or *CFAP52* mutations do not fulfill the diagnostic criteria for PCD, due to no indication of neonatal respiratory distress syndrome or chronic destructive lung disease (bronchiectasis) as well as nNO production rate levels generally above the currently accepted cutoff (77 nl / min.) for PCD referral cases[1,2]. All seven individuals present LRA abnormalities including *situs inversus totalis*. CFAP45-deficient individual OP-28 II1 shows no evidence of bronchiectasis by chest CT scan (**Supplementary Fig. 5p**) but reported mild respiratory symptoms (rhinosinusitis and bronchitis) as well as recurrent effusions of the middle ear (otitis media). Asthenospermia is also indicated (see also **Supplementary Videos 1 and 2** and **Fig. 1**). CFAP52-deficient individual OI-81 II1 shows no evidence of bronchiectasis by chest CT scan (**Supplementary Fig. 5q**) but reported asthma-like respiratory symptoms and otitis media as well as infertility. Spirometry values for OI-81 II1: FVc, 111%, FEV1, 109%, and FeF25-75, 88% with no significant reversibility. Bronchiectasis is almost universal by adulthood in PCD individuals[1,2]. Ta-Shma et al.[3] also reported normal nNO levels (578 ppb or 174 nl / min.), normal ciliary structure and no indication of bronchiectasis in unrelated siblings with loss-of-function *CFAP52* mutations. n.r. = not reported. See also **Supplementary Fig. 1** and **Methods**.

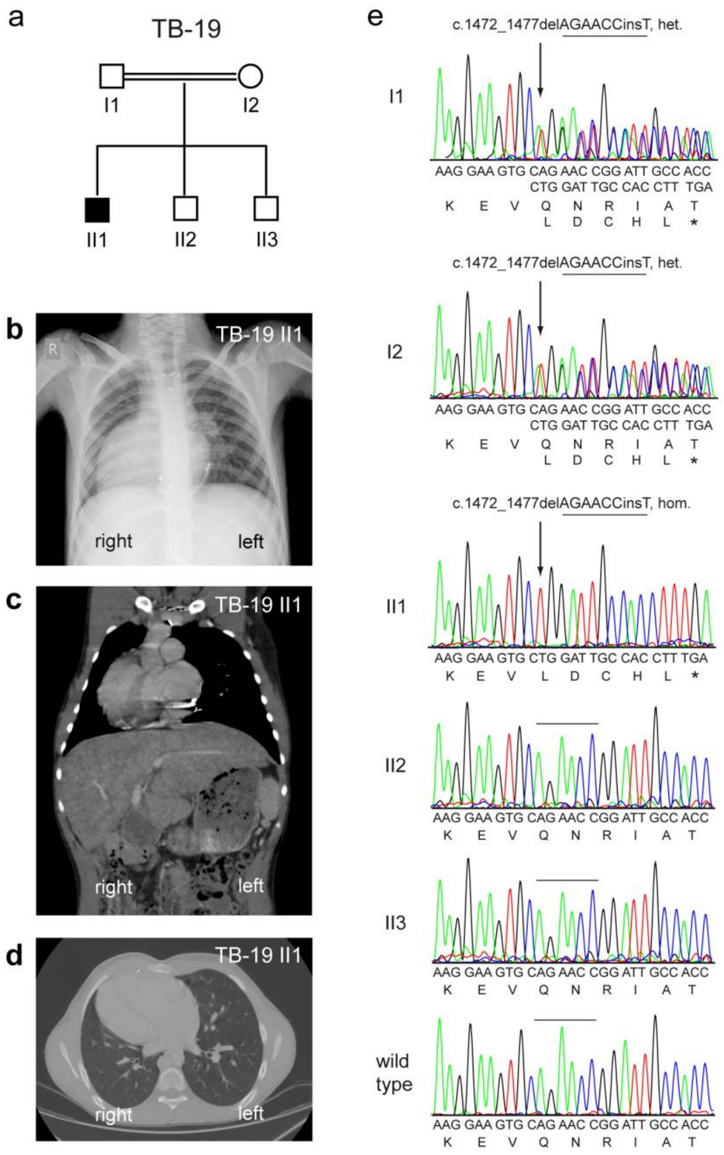

Supplementary Fig. 1 | WES identifies a homozygous frameshift *CFAP45* variant in individual TB-19 II1 diagnosed with heterotaxy syndrome. **a**, Family TB-19 is a consanguineous mating with one affected male individual (TB-19 II1) and two healthy male individuals (TB-19 II2, TB-19 II3. **b**, Chest X-ray of TB-19 II1 shows dextrocardia with heart on the right side; TB-19 II1 was prenatally diagnosed with a heart defect (large primum-type atrial septal defect and partial atrio-ventricular canal defect) that required corrective surgery at 6 months of age. **c**, TB-19 II1 also presented duodenal atresia (surgically repaired at 3 weeks of age), intestinal malrotation and polyspenia, consistent with heterotaxy syndrome. **d**, Chest CT scan of TB-19 II1 at 16 years of age shows no evidence of bronchiectasis. **e**, Sanger sequencing verifies homozygous *CFAP45* variant c.1472_1477delAGAACCinsT in affected individual TB-19 II1; both parents are heterozygous for this variant and healthy male siblings TB-19 II2 and TB-19 II3 are comparable to wild type. This *CFAP45* variant in exon 11 results in a frameshift and premature termination of translation (p.Gln491Leufs*5). Light microscopy analysis of respiratory cells obtained by nasal brush biopsy indicated normal motility; nasal nNO (337.2 nl / min.) is above the value for PCD referral cases (77 nl / min.). See also **Supplementary Table 1** and **Supplementary Table 2**.

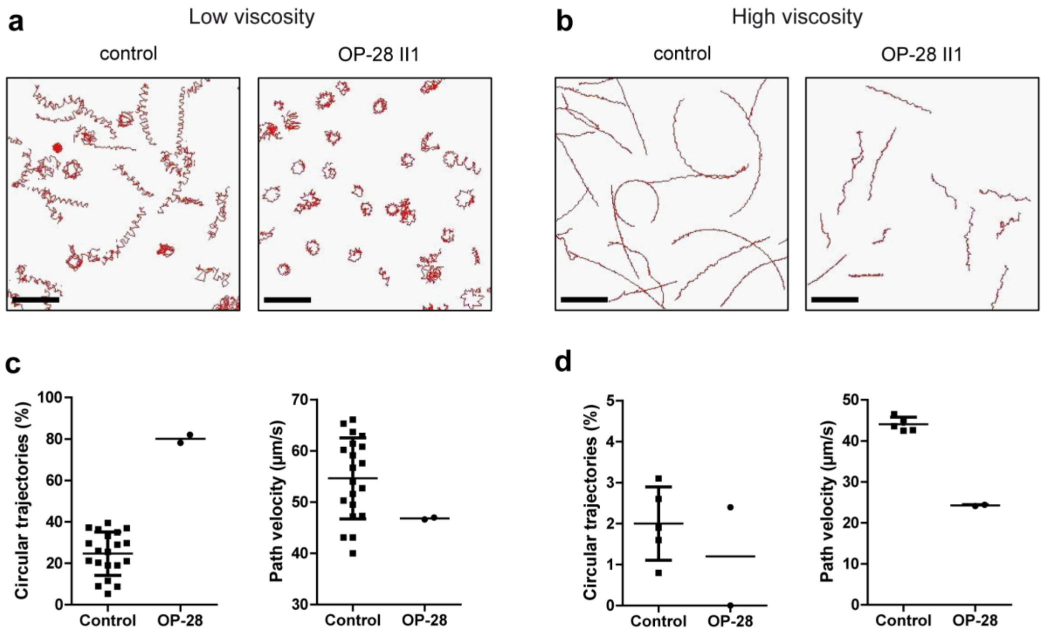

Supplementary Fig. 2 | CFAP45-deficient human sperm exhibit circular swimming in low viscosity media and reduced path velocity in high viscosity media. a,**b**, Representative swimming paths of healthy control and CFAP45-deficient sperm from individual OP-28 II1 in standard HTF+++ medium (low viscosity) (**a**) and viscous HTF+++ medium supplemented with 1% (w/v) methyl cellulose (high viscosity) (**b**). Scale bars represent 50 µm.

c, Compared to healthy control in low viscosity media, the percentage of circular trajectories of CFAP45-deficient sperm from individual OP-28 II1 is more than two-fold higher (25 ± 10% vs. 80%, respectively), whereas the average path velocity between the two groups is comparable (55 ± 8 µm / s vs. 47 µm / s, respectively). Data are the mean ± SD; control, *n* = 20; OP-28 II1, *n* = 1 (two independent measurements from one sperm sample). **d**, Compared to healthy control in high viscosity media, the percentage of circular trajectories of CFAP45-deficient sperm from individual OP-28 II1 is similar (2.0 + 0.9% vs. 1.2%, respectively) but their average path velocity is approximately 45% slower (44 ± 2 µm / s vs. 24 µm / s, respectively). Data are the mean ± SD; control, *n* = 5; OP-28 II1, *n* = 1 (two independent measurements from one sperm sample). See also **Methods**.

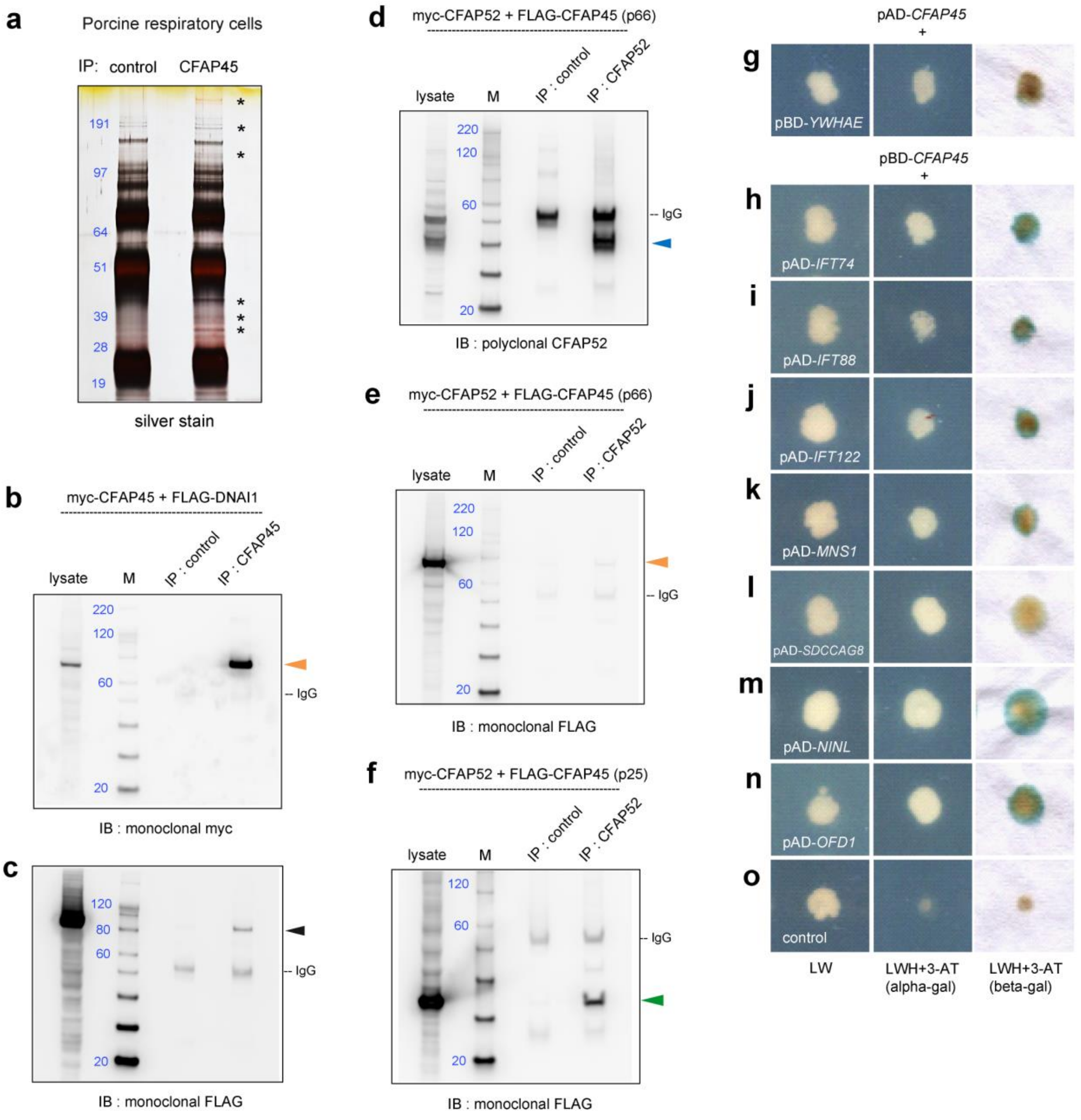

Supplementary Fig. 3 | CFAP45 associations identified by immuno-affinity capture and LC/ MS-MS or Y2H. **a**, Representative silver stained-gel of CFAP45 immunoprecipitate from porcine respiratory lysate (*n* = 1). Asterisks highlight bands detectable in CFAP45 but not control immunoprecipitates. Blue numerals indicate relative molecular weight (kDa). See **Supplementary Data 1** for full summary of associations. **b**-**f**, Validation of CFAP45 associations with DNAI1 and CFAP52 in HEK293 cells. In contrast to control, polyclonal CFAP45 antibody immunoprecipitates myc-tagged CFAP45 (p66) (orange arrowhead) (**b**) and FLAG-tagged DNAI1 (black arrowhead) (*n* = 2) (**c**). In contrast to control, polyclonal CFAP52 immunoprecipitates myc-tagged CFAP52 (blue arrowhead) (**d**) and FLAG-tagged CFAP45 (p25) (green arrowhead) (**f**) but not FLAG-tagged CFAP45 (p66) (orange arrowhead) (*n* = 3) (**e**). CFAP45 isoform (p25) lacks the Trichoplein domain and utilizes an alternative carboxyl terminus (see also **Fig. 1j** and **Fig. 5o** and **Methods**). Recombinant CFAP52, CFAP45 (p66), CFAP45 (p25) and DNAI1 are 42, 74, 33 and 83 kilodaltons, respectively. Marker (M) indicates relative molecular weight (blue numerals) in kilodaltons. **g**-**o**, Y2H of full-length CFAP45 (p66) and select “ciliaproteome“ proteins (see also **Methods**). Positive interactions are indicated by growth on LWH+3-AT media (**g**-**n**), in contrast to negative control (**o**). CFAP45 (p25) was not tested by Y2H.

ADGRV1	CEP104	DYNLRB2	IFT57	NINL	RPGRIP1L	TUBA1A
AHI1	CEP164	DYNLT1	IFT74	NME7	RSPH9	TUBB4B
AK7	CEP290	DZIP1	IFT81	NME8	SDCCAG8	TULP1
ARL13B	CETN2	E2F4	IFT88	NPHP4	SMU1	TULP3
ARL3	CETN3	EFHC1	INPP5E	OFD1	SOGA1	UBE2K
ARL6	CFAP161	EFHC2	INTU	PACRG	SPAG16	UBE2N
ARMC8	CFAP20	EPS8	INVS	PAFAH1B1	SPAG5	UNC119
B9D1	CFAP52	ESPN	IQCA1	PKD2L1	SPAG6	USH1C
B9D2	CFAP58	FOXJ1	IQCB1	POLL	SPATA4	USH2A
BBS1	CFAP61	FUZ	IQUB	PPP1CA	SPATA7	VANGL2
BBS10	CFAP91	GID8	KIF3A	PPP1CB	SPEF1	VHL
BBS12	CLUAP1	GLIS3	KIF3B	PPP1R7	SSNA1	VIM
BBS2	CMPK1	GSTP1	LCA5L	PPP2R1A	STEAP3	WDR19
BBS4	CRB1	HSF1	LRRC6	PRDX2	STK36	WDR26
BBS5	CRB2	HSPA1L	LZTS2	PTPDC1	STOM	WDR34
BBS7	CYB5D1	HSPA5	MAEA	RAB3IP	TBX6	WDR35
BBS9	DCTN1	HSPB11	MAPRE1	RAB8A	TEKT2	WDR47
C8orf37	DCTN2	IFT122	MAPRE2	RANBP10	TMEM216	WDR60
CACNA1D	DNAI1	IFT140	MKKS	RFX3	TRAF3IP1	WDR66
CALM2	DNAI2	IFT20	MKLN1	RIBC1	TRIM32	WDR78
CAMK2A	DNALI1	IFT22	MNS1	RMND5A	TTC21A	WHRN
CBY1	DPP3	IFT27	NEK1	RMND5B	TTC30A	XPNPEP3
CC2D2A	DPY30	IFT43	NEK4	RP2	TTC30B	YPEL5
CCDC65	DYNC2LI1	IFT46	NEK7	RPGR	TTC8	YWHAE
CDR2	DYNLL2	IFT52	NEK8	RPGRIP1	TTLL1	ZMYND12

Supplementary Table 3 | List of genes tested for binary interactions with CFAP45 by Y2H. Partial and / or full length constructs of 175 genes (NCBI gene symbol) were screened 1:1 for binary interaction to CFAP45 by filter lift yeast two-hybrid analysis.

Supplementary Data 1 | Curation of associations identified for CFAP45 and CFAP52. Associations identified for CFAP45 and CFAP52 were further curated for likely ciliary function, tissue distribution, nucleotide binding, and links to asthenospermia. Ciliary function is inferred by *in silico*[4,5] or proteomic[6] studies; tissue distribution indicates RNA and protein expression profile provided by The Human Protein Atlas database; nucleotide binding indicates annotation in the DAVID Bioinformatics database or identification by Adachi et al[7]; asthenospermia indicates proteins that were downregulated in asthenospermic males in the study by Amaral et al[8]. Please see **Supplementary Data 1** for the full list of curated associations.

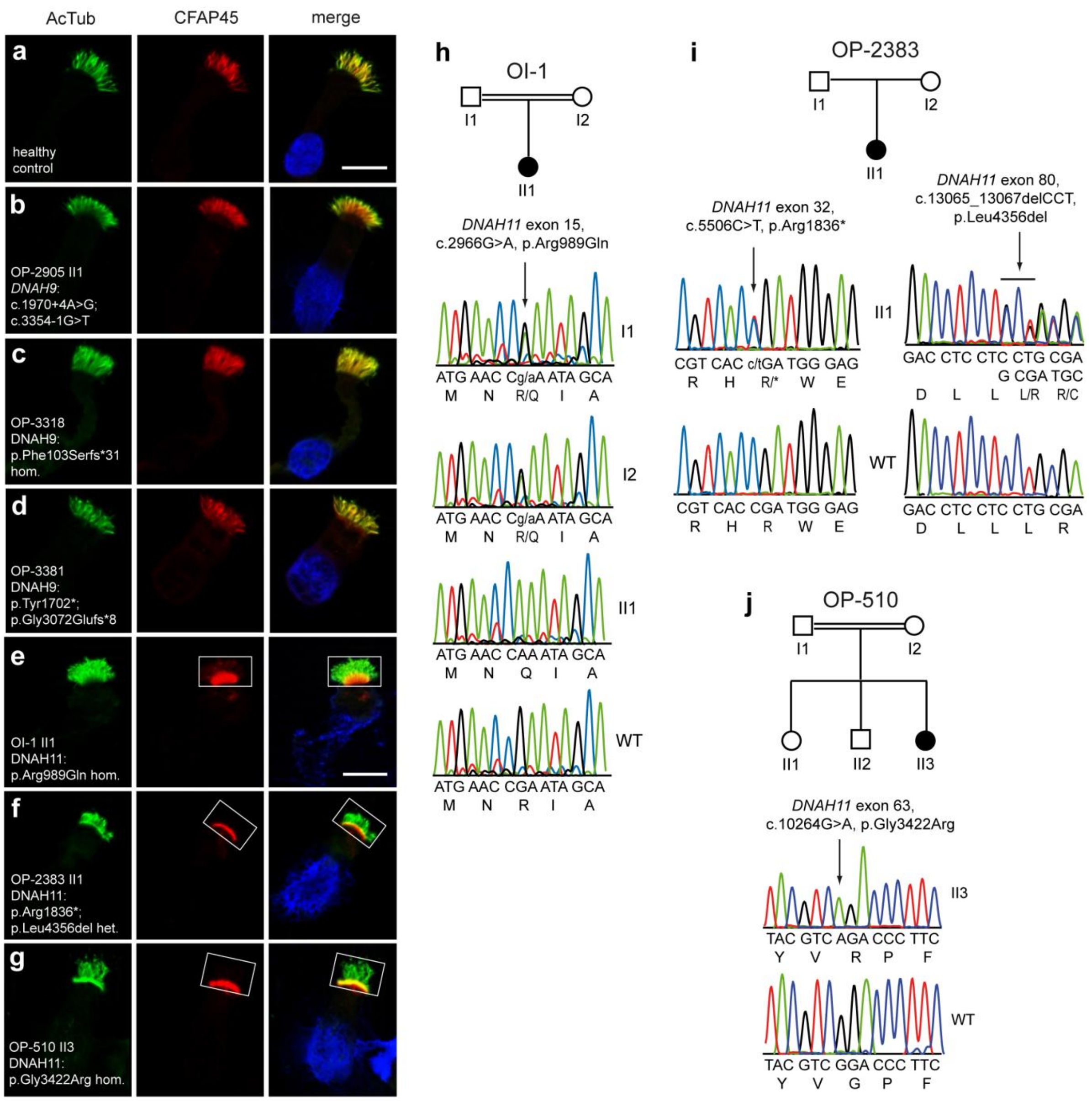

Supplementary Fig. 4 | Ciliary CFAP45 localization is abnormal in certain *DNAH11*-mutant but not *DNAH9*-mutant cilia by IFM. a-d, Similar to healthy control respiratory cilia (**a**), CFAP45 (red) is panaxonemal in *DNAH9*-mutant respiratory cilia. Individual OP-2905 II1 (**b**) with compound heterozygous *DNAH9* variants c.1970+4A>G and c.3354-1G>T has been described[9]. OP-3318 (**c**) (*DNAH9*, c.308delT, p.Phe103Serfs*31 hom.) and OP-3381 (**d**) (*DNAH9*, c.5106T>G, p.Tyr1702*, het.; c.9211_9214dupAACG, p.Gly3072Glufs*8, het.) show autosomal recessive inheritance by homozygosity or heterozygosity, respectively. Respiratory cilia from *DNAH9*-mutant individuals show complete loss of ODAs (DNAH5 and DNAH9) in the distal ciliary compartment; DNAH11 retains wild type proximal ciliary immunolocalization in *DNAH9*-mutant respiratory cilia. **e-f**, CFAP45 (red) is detectable at the ciliary base but not in axonemes (rectangles) of certain *DNAH11*-mutant respiratory cilia from individual OI-1 II1 (**e**), OP-2383 II1 (**f**), and OP-510 II3 (**g**). Ciliary axonemes (green) are detected using anti-acetylated α tubulin (AcTub) antibody. Merge images include Hoechst stain (blue) to indicate nuclei. White scale bar equals 10 µm. **a-g**, n = 14 images from 2 experiments. **h-j**, Sanger sequencing verifies *DNAH11* mutations in OI-1 II1 (**h**) (c.2966G>A, p.Arg989Gln, hom.), OP-2383 II1 (**i**) (c.5506C>T, p.Arg1836*, het.; c.13065_13067delCCT, p.Leu4356del, het.), and OP-510 II3 (**j**) (c.10264G>A, p.Gly3422Arg). All three individuals were diagnosed with

PCD and showed a phenotype consistent with *DNAH11* mutations, including hyperkinetic beating (OI-1 II1 and OP-2383 II1) and normal ciliary ultrastructure by TEM (OI-1 II1 and OP-2383 II1).

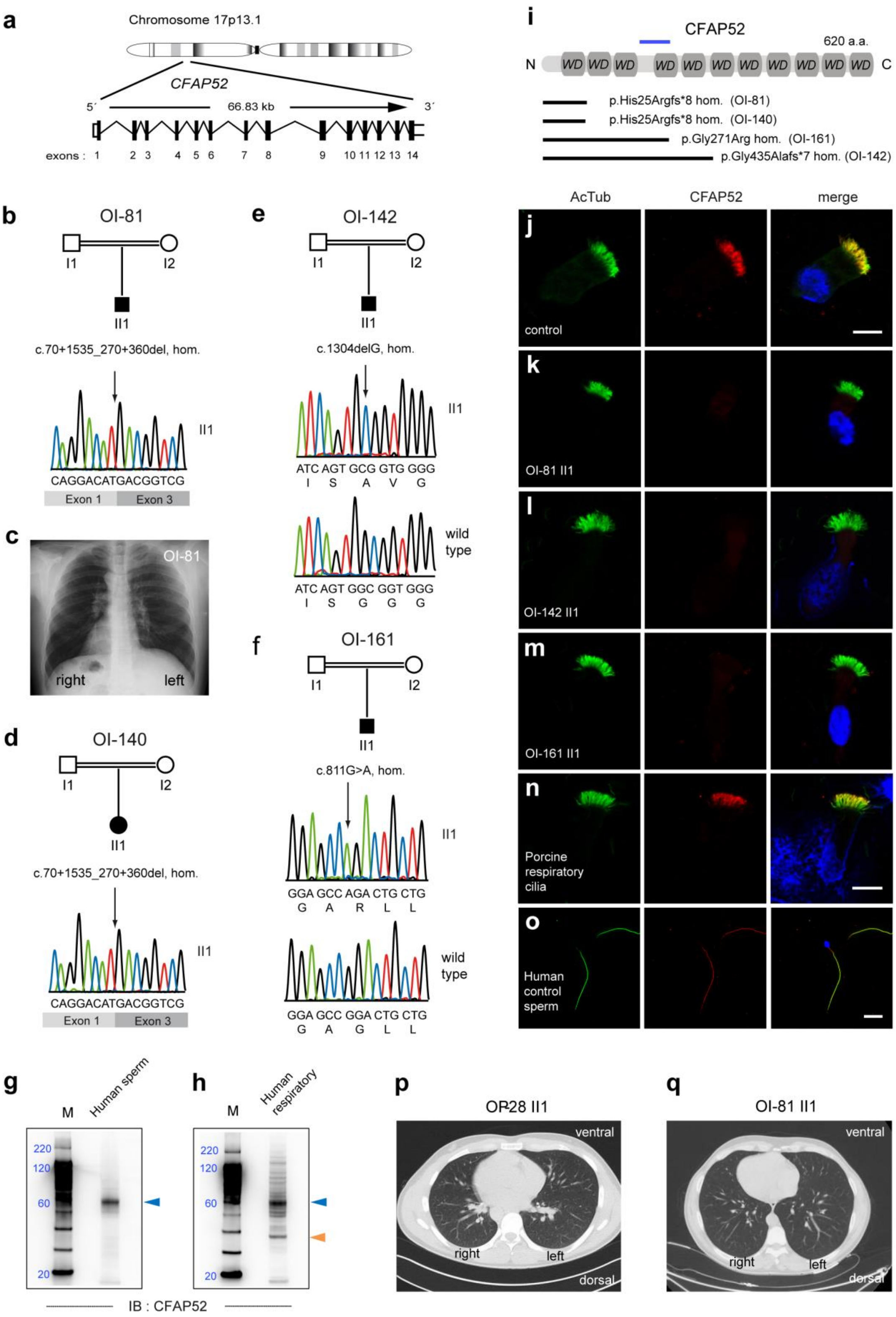

Supplementary Fig. 5 | *CFAP52* mutations cause a motile ciliopathy. a-**f**, Loss-of-function mutations in *CFAP52* (**a**) including homozygous *CFAP52* frameshift mutations (exon 2 deletion) in unrelated individuals OI-81 (**b**) with *situs inversus totalis* (indicated by X-ray scan) (**c**) and OI-140 (**d**), a homozygous frameshift mutation in individual OI-142 (**e**), and homozygous missense mutation in individual OI-161 (**f**). **g**,**h**, IB detects a CFAP52 isoform of approximately 60 kDa (blue arrowhead, 552 amino acids) in human sperm (**g**) and respiratory (**h**) lysates (*n* = 2 for each tissue). A smaller CFAP52 isoform (orange arrowhead) is detectable in respiratory lysates. Marker (M)

indicates relative molecular weight (blue numerals) in kilodaltons. **i**, Overview of CFAP52 protein, which contain WD40-repeat domains, indicating protein change of CFAP52 mutations; blue line indicates relative epitope position of anti-CFAP52 antibody (amino acids 220 – 313). **j**-**o**, In contrast to control (**j**), panaxonemal CFAP52 localization (red) is undetectable in respiratory cilia from individuals OI-81 (**k**), OI-142 (**l**) and OI-161 (**m**) by IFM. This agrees with the predicted loss-of-function frameshift mutations in individuals OI-81, OI-140 and OI-142. Individual OI-161 harbors the missense variant rs140921334 that is predicted to be deleterious (SIFT) or probably damaging (Polyphen-2); although we cannot rule out that this amino acid exchange (p.Gly271Arg) affects epitope recognition of the anti-CFAP52 antibody (**i**), this variant is likely pathogenic (see **Supplementary Fig. 8i**). **n**,**o**, CFAP52 (red) is detectable in porcine respiratory (**n**) and human sperm flagellar (**o**) axonemes. Ciliary and flagellar axonemes (green) are detected using anti-acetylated α tubulin (AcTub) antibody. Merge images include Hoechst stain (blue) to indicate nuclei. White scale bar equals 10 μm. **j**-**o**, *n* = 18 images over 3 experiments. **p**,**q**, Chest CT scans do not indicate bronchiectasis in CFAP45-deficient individual OP-28 II1 (31 years of age) (**p**) or CFAP52-deficient individual OI-81 II1 (51 years of age).

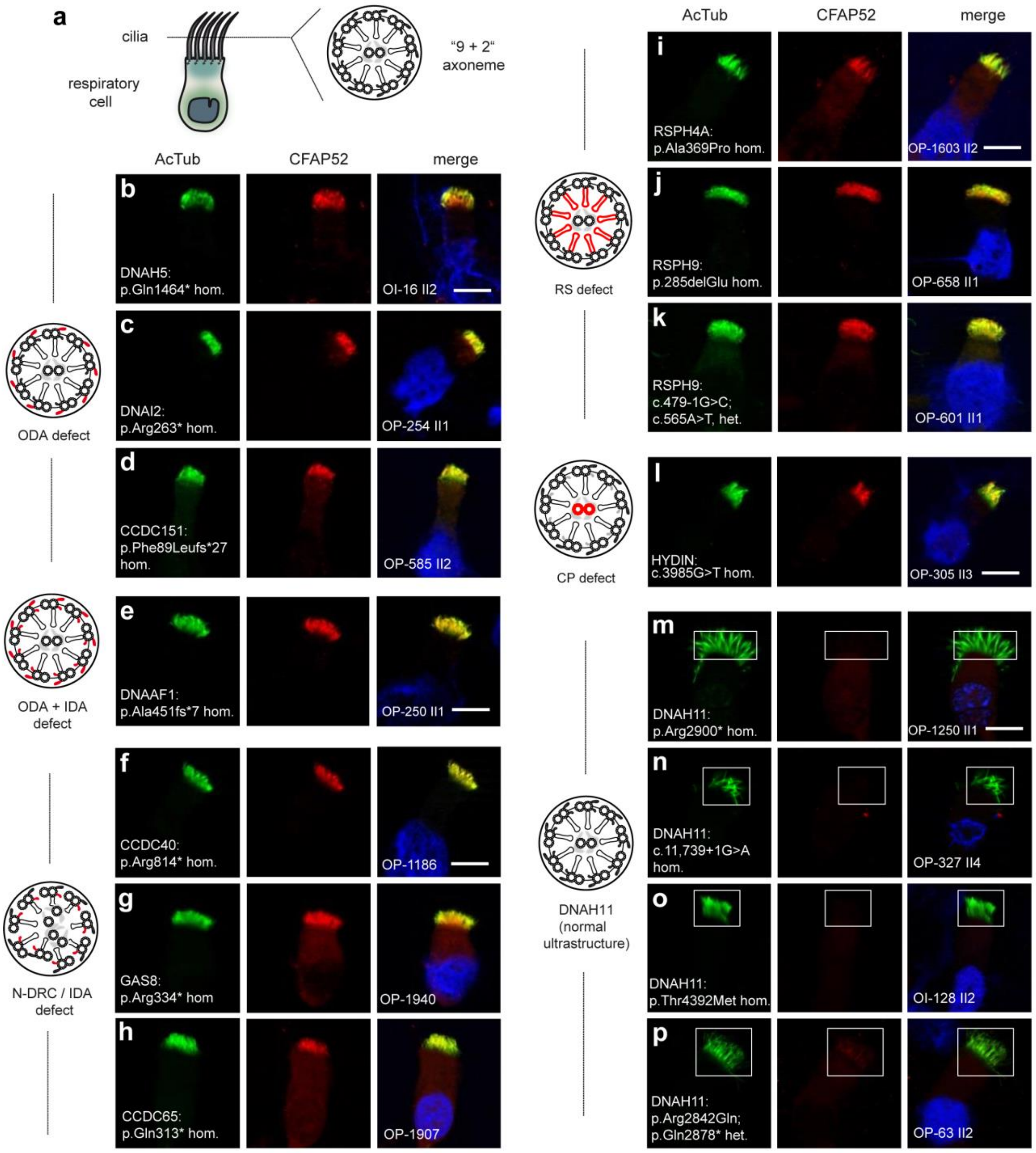

Supplementary Fig. 6 | Ciliary CFAP52 localization is abnormal in certain DNAH11-mutant cilia by IFM.
a, Cartoon of “9+2” axoneme in respiratory cilia. Axonemal defects that correlate with certain PCD-causing mutations are highlighted in red. **b**-**d**, Isolated ODA defects in *DNAH5*-(**b**), *DNAI2*-(**c**), and *CCDC151*-mutant (**d**) respiratory cilia do not affect ciliary CFAP52 (red) localization. **e**, Compound ODA and IDA defects in *DNAAF1*-mutant respiratory cilia do not affect ciliary CFAP52 (red) localization. **f**-**h**, N-DRC defects with (*CCDC40*-mutant respiratory cilia) (**f**) or without IDAs and tubular disorganization (*GAS8*- and *CCDC65*-mutant respiratory cilia) (**g**,**h**, respectively) do not affect ciliary CFAP52 (red) localization. **i**-**k**, RS defects in *RSPH4A*- and *RSPH9*-mutant respiratory cilia do not affect ciliary CFAP52 (red) localization. **l**, CP defects in *HYDIN*-mutant respiratory cilia do not affect CFAP52 (red) localization. **m**-**p**, Certain *DNAH11*-mutant respiratory cilia show abnormal ciliary CFAP52 (red) localization (rectangles). Ciliary axonemes (green) are detected using anti-acetylated α tubulin (AcTub) antibody. Merge images include Hoechst stain (blue) to indicate nuclei. White scale bar equals 10 µm. **b**-**p**, n = 24 images over 4 experiments. PCD individuals (merge panel) were reported previously.

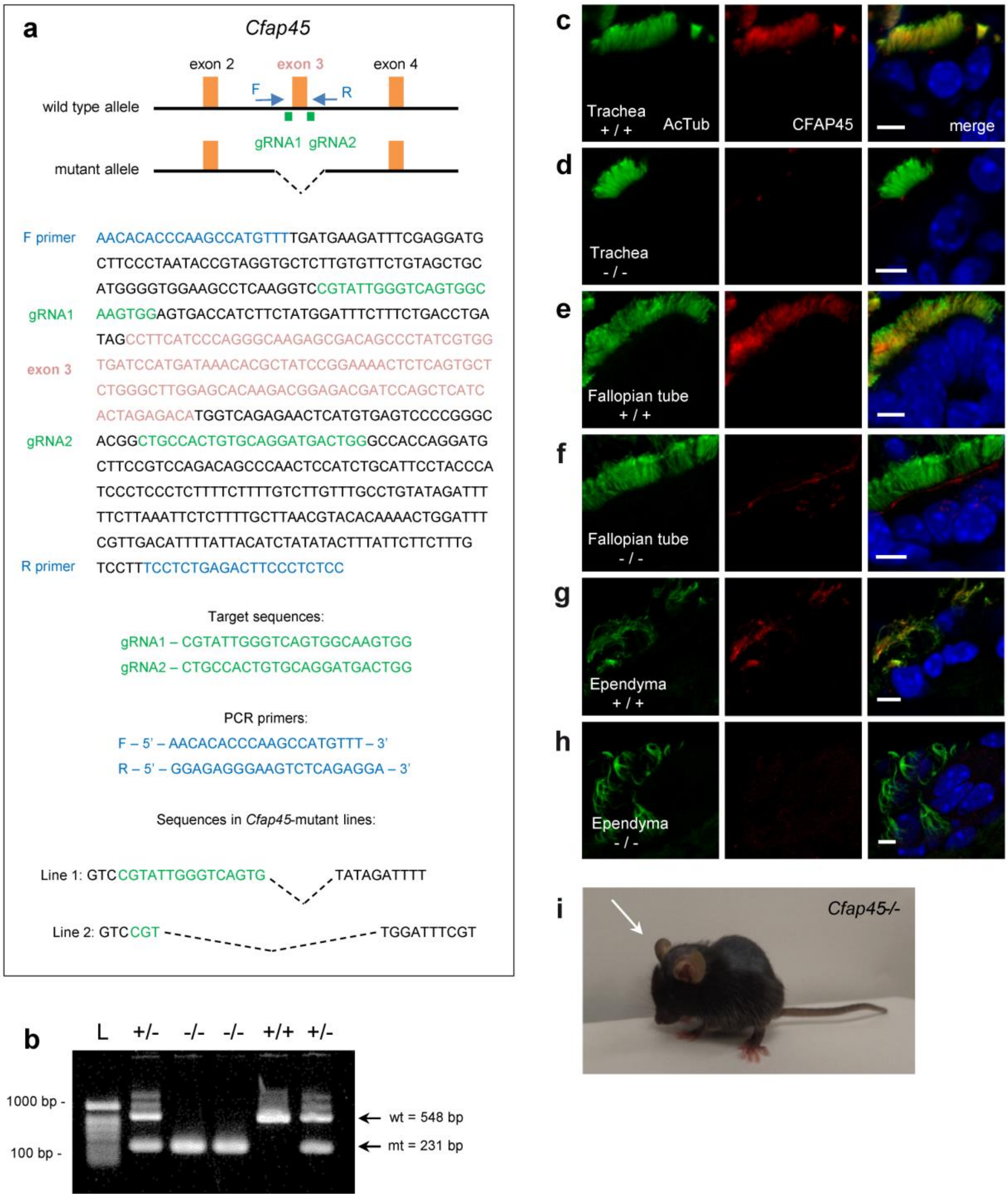

Supplementary Fig. 7 | Generation and validation of *Cfap45*$^{-/-}$ mice. a, Schematic of CRISPR / Cas9-based deletion of exon 3 in *Cfap45* (ENSMUST00000085894.11), which is located on mouse chromosome 1 and encodes a full-length 551 amino acid protein (Uniprot Q9D9U9) with 83% identity to human CFAP45. **b**, PCR (n = 13) demonstrates that in contrast to wild type (wt, +/+) and heterozygous (+/-) controls, mutant (mt, -/-) *Cfap45*$^{-/-}$ mice lack exon 3. L = ladder; bp = base pairs. **c**-**d**, CFAP45 is detectable by IFM in respiratory cilia of trachea in wild type (+/+) but not *Cfap45*$^{-/-}$ mice. **e**-**f**, CFAP45 is detectable by IFM in multiciliated epithelia of Fallopian tube in wild type (+/+) but not *Cfap45*$^{-/-}$ mice. **g**-**h**, CFAP45 is detectable by IFM in multiciliated ependyma of brain in wild type (+/+) but not *Cfap45*$^{-/-}$ mice. Samples were costained with anti-acetylated α tubulin (AcTub) antibody to visualize ciliary axonemes (green). Merge images include Hoechst stain to visualize nuclei (blue). White scale bars equal 5 µm. **c**-**h**, n = 12 images from 2 experiments for each tissue. **i**, *Cfap45*$^{-/-}$ mice present with hydrocephalus (white arrow) with a characteristic dome-shaped crown. CFAP45- and CFAP52-deficient individuals in this study do not present hydrocephalus.

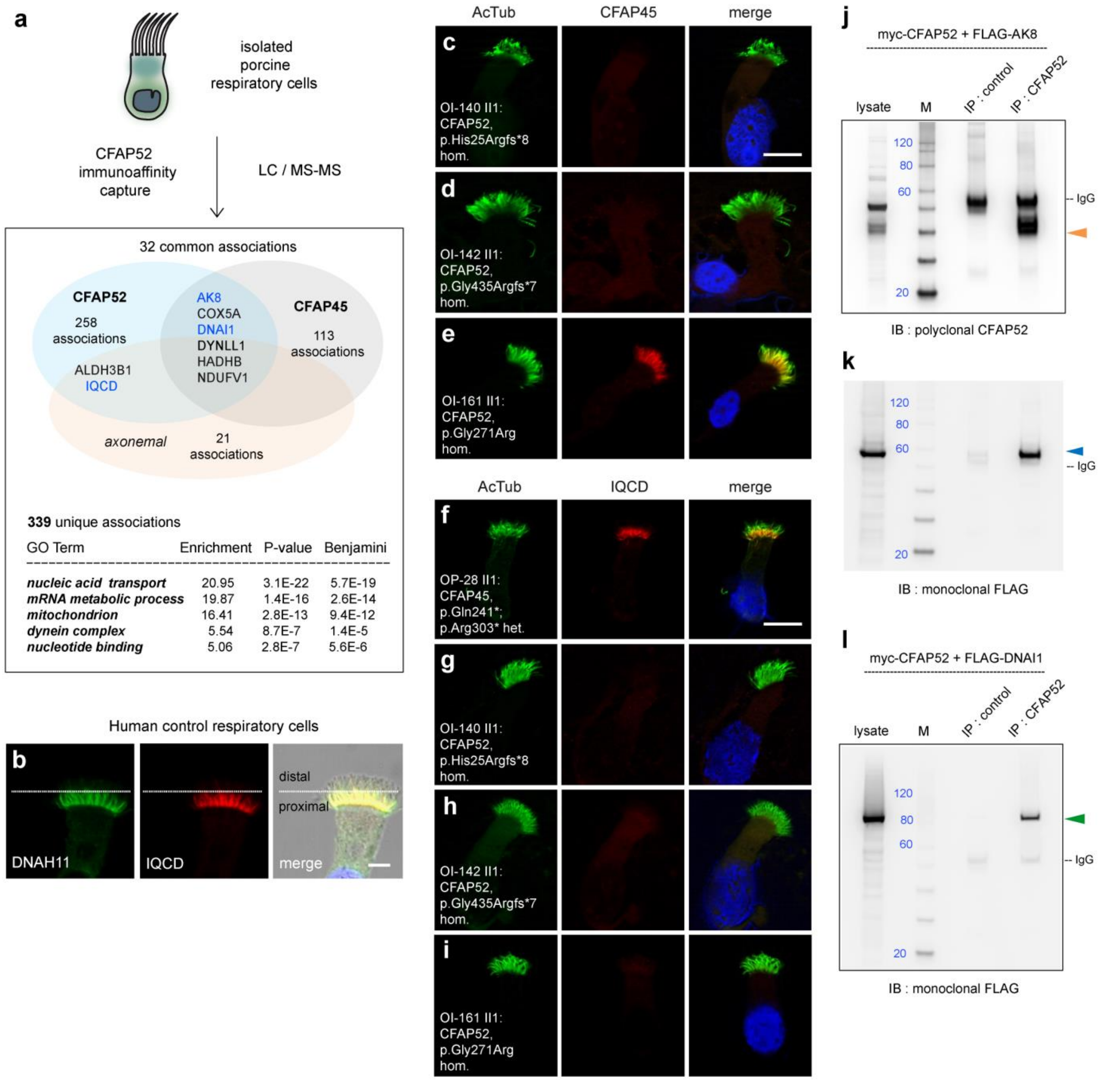

Supplementary Fig. 8 | Ciliary CFAP52 associations identified by immuno-affinity capture and LC/ MS-MS.
a, CFAP52 immunoprecipitates from porcine respiratory cells identify 258 interactors by MS including the N-DRC-associated protein IQCD; 16 proteins including CFAP52 were identified all three times.. Curation of combined respiratory cell MS hits for CFAP45 and CFAP52 reveal 32 common associations including AK8 and DNAI1 with enrichments linking metabolic processes to dynein complex (see also **Supplementary Data 1**). **b**, IQCD localizes to the proximal region of respiratory cilia, reminiscent of DNAH11. IQCD (red) colocalizes with DNAH11 (green) in the proximal ciliary region of human control respiratory cells. Merge panel includes differential interference contrast to highlight proximal and distal ciliary regions (white lines). *n* = 10 images from 2 experiments. **c**-**i**, CFAP52 homozygous missense variant pGly271Arg affects ciliary localization of IQCD but not CFAP45. In contrast to *CFAP52*-deficient respiratory cilia from OI-140 (**c**) and OI-142 (**d**), CFAP45 (red) is still detectable in *CFAP52*-deficient respiratory cilia from individual OI-161 with homozygous missense variant pGly271Arg (**e**). In contrast to CFAP45-deficient respiratory cilia (**f**), IQCD (red) is undetectable in *CFAP52*-deficient respiratory cilia from individuals OI-140 (**g**) and OI-142 (**h**) as well as OI-161 (**i**). This further validates the pathogenicity of missense variant pGly271Arg in OI-161 (**Supplementary Fig. 5f and m**) and demonstrates that CFAP52 deficiency can affect both CFAP45 and IQCD ciliary localization. Ciliary axonemes (green) are detected using anti-acetylated α

tubulin (AcTub) antibody. Merge images include Hoechst stain (blue) to indicate nuclei. White scale bars equal 10 μm. **c**-**i**, *n* = 14 images from 2 experiments. **j**-**l**, Validation of CFAP52 associations with AK8 and DNAI1 in HEK293 cells. In contrast to control, polyclonal CFAP52 antibody immunoprecipitates myc-tagged CFAP52 (orange arrowhead) (**j**) and both FLAG-tagged AK8 (blue arrowhead) (**k**) and FLAG-tagged DNAI1 (green arrowhead) arrowhead) (**l**). Recombinant CFAP52, AK8 and DNAI1 are 42, 60 and 83 kilodaltons, respectively. Marker (M) indicates relative molecular weight (blue numerals) in kilodaltons. **j**-**l**, *n* = 2.

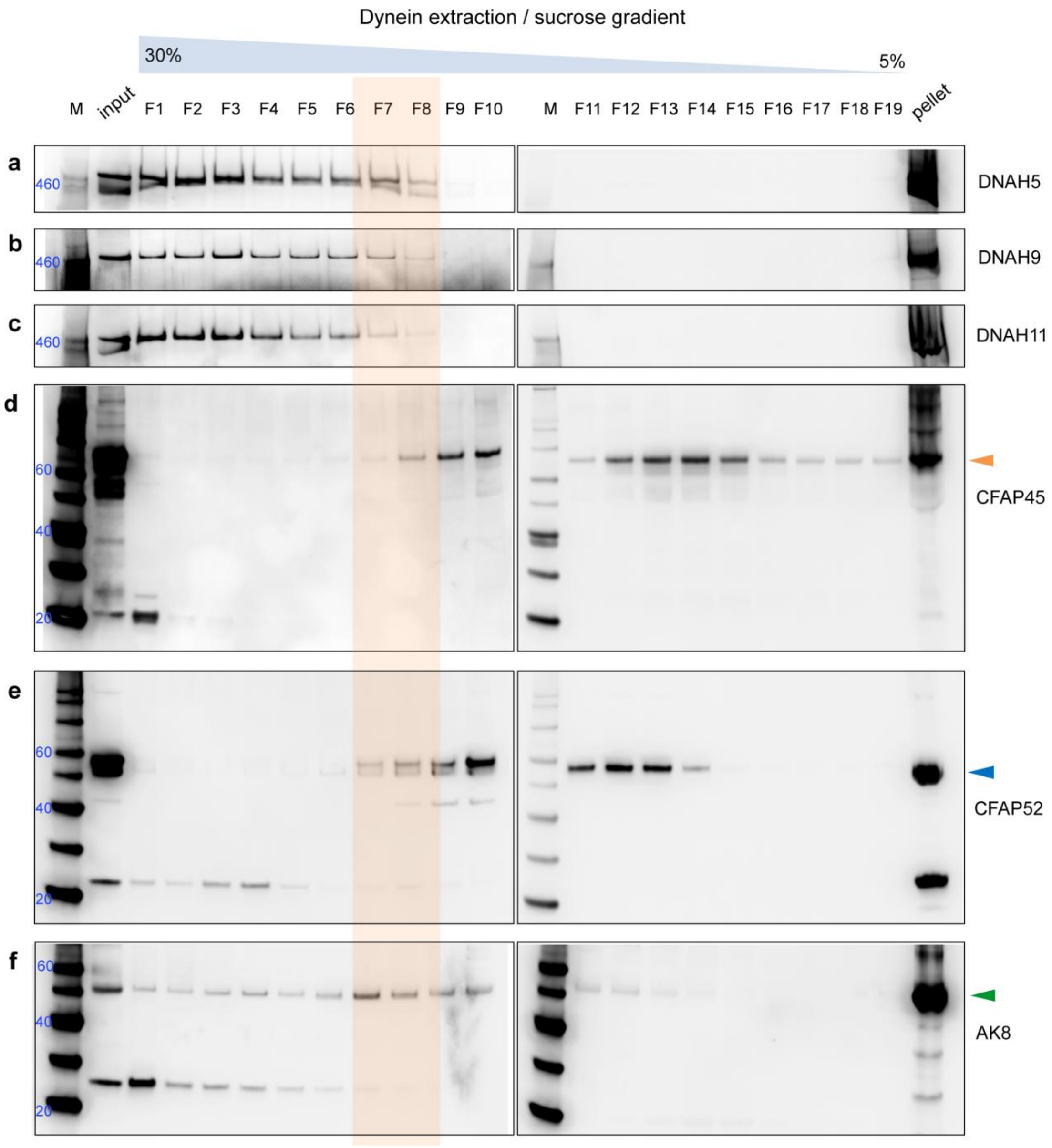

Supplementary Fig. 9 | CFAP45, CFAP52 and AK8 cofractionate with ODA components including DNAH11. Dynein ATPases were extracted from porcine respiratory cilia as described[10] and fractionated across a 5-30% sucrose gradient. **a-c**, ODA dynein ATPases DNAH5 (**a**), DNAH9 (**b**), and DNAH11 (**c**) are detectable in F1-F8 by IB. **d-f**, CFAP45, CFAP52 and AK8 cofractionate with DNAH11 in F7-F8. Full length CFAP45 (orange arrowhead) is detectable in DNAH11-positive fractions F7 and F8 by IB (**d**). Full length CFAP52 (blue arrowhead) is detectable in DNAH11-positive fractions F7 and F8 by IB (**e**). Full length AK8 (green arrowhead) is detectable in DNAH11-positive fractions F7 and F8 by IB (**f**). Input represents supernatant enriched for dynein ATPases before sucrose gradient fractionation; 15 µl of a 2.0% by volume aliquot is analyzed. Fractions represent 600 µl of an 11.5 ml dynein extract / sucrose suspension; 15 ul of a 3.5% by volume aliquot is analyzed. Pellet represents post-supernatant sample resuspended in 500 µl of 1X LDS sample buffer; 15 µl of an 8-fold enrichment by supernatant volume aliquot is analyzed. Rabbit polyclonal antibodies to DNAH5, DNAH9, DNAH11, CFAP45, CFAP52, and AK8 are used at a final concentration of 0.25 µg / ml. Porcine DNAH5, DNAH9, DNAH11, CFAP45, CFAP52, and AK8 are 528, 510, 519, 66.5, 61.5 and 46 kilodaltons, respectively. Marker (M) indicates relative molecular weight (blue numerals) in kilodaltons. Full blot images of DNAH5, DNAH9, and DNAH11 are provided in **Source Data** file. **a**-**f**, *n* = 2. See also **Methods**.

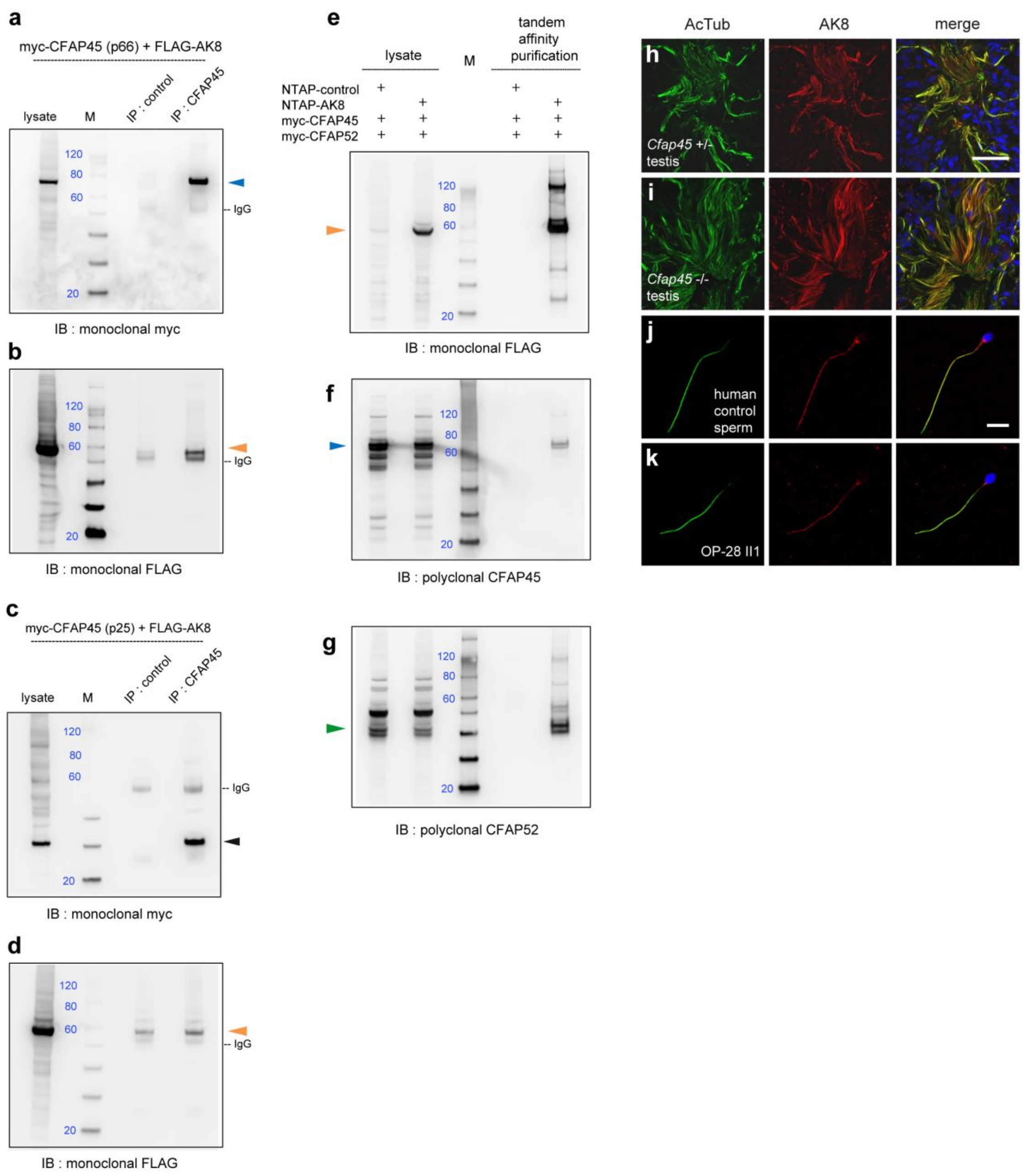

Supplementary Fig. 10 | AK8 interacts with CFAP45 and CFAP52. a-d, AK8 differentially associates with CFAP45 isoforms in HEK293 cells. In contrast to control, polyclonal CFAP45 antibody immunoprecipitates myc-tagged CFAP45 (p66) (blue arrowhead) (**a**) and FLAG-tagged AK8 (orange arrowhead) (**b**). In contrast to control, polyclonal CFAP45 antibody immunoprecipitates myc-tagged CFAP45 (p25) (black arrowhead) (**c**) but not FLAG-tagged AK8 (orange arrowhead), which shows comparable intensity to control (**d**). **a**-**d**, n = 2. **e**-**g**, In contrast to control, tandem affinity purification of AK8 (orange arrowhead) from HEK293 cells (**e**) co-elutes both CFAP45 (p66) (blue arrowhead) (**f**) and CFAP52 (green arrowhead) (**g**). **e**-**g**, n = 3. Recombinant CFAP52 (green arrowhead), CFAP45 (p66) (blue arrowhead), CFAP45 (p25) (black arrowhead) and AK8 (orange arrowhead) are 42, 74, 33 and 60 kilodaltons, respectively. Marker (M) indicates relative molecular weight (blue numerals) in kilodaltons. **h**-**k**, AK8 retains wild type localization in CFAP45-deficient mouse and human sperm flagella. Similar to heterozygous control (**h**), AK8 (red) is detectable in *Cfap45*$^{-/-}$ testis (**i**). Similar to normospermic control (**j**), AK8 (red) is detectable in sperm flagella of CFAP45-deficient individual OP-28 II1 (**k**). Samples are counterstained for

ciliary axonemes (green) using anti-acetylated α tubulin (AcTub) antibody. Merge images include Hoechst stain to visualize nuclei (blue). White scale bars equal 10 µm. **h**-**k**, *n* = 12 images from 2 experiments.

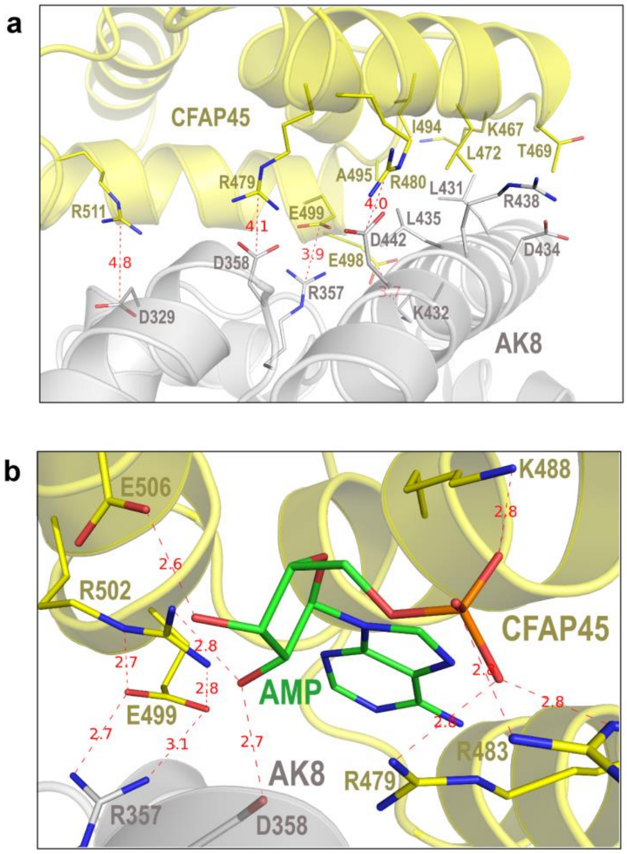

Supplementary Fig. 11 | Interface of CFAP45•AMP•AK8 complex. a, Close-up view at the interface of CFAP45•AMP•AK8. AMP is undisplayed for clarity. The five intermolecular salt bridges at the interface are marked with thin-dash lines. Distances shown by thin-dash lines are in Angstroms (Å). The side chains of T469, L472, I494, K467, and A495 in CFAP45 have Van der Waal interactions at the interface with the side chains of L431, D434, L435, and R438 in AK8. **b**, Close-up view of AMP bound at the interface of CFAP45•AK8 complex. Carbon atoms in AMP, CFAP45, and AK8 are colored in green, yellow, and grey, respectively; nitrogen, oxygen, and phosphorus atoms are colored in blue, red, and orange, respectively. Distances shown by thin-dash lines are in Angstroms (Å).

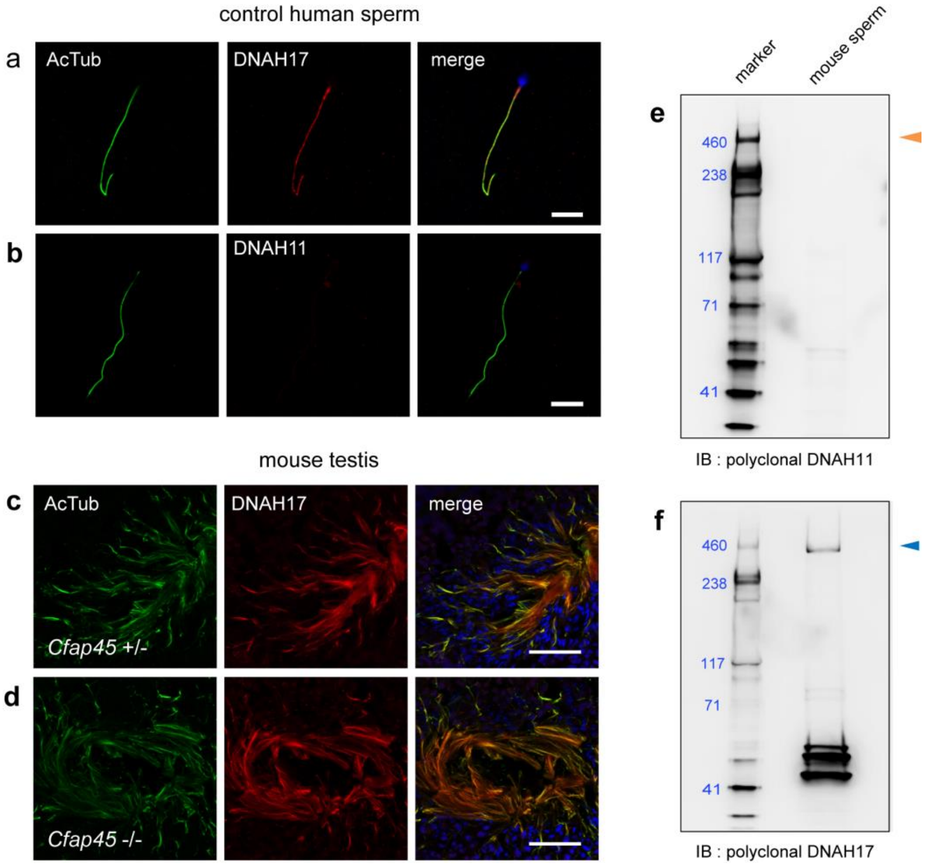

Supplementary Fig. 12 | DNAH17 but not its paralog DNAH11 is detectable in mammalian sperm flagella. **a**, DNAH17 (red) is detectable in the flagella of healthy control human sperm by IFM. **b**, DNAH11 (red) is not detectable in the flagella of healthy control human sperm by IFM. **a**,**b**, *n* = 18 images from 3 experiments. **c**,**d**, DNAH17 (red) is detectable in both CFAP45 heterozygous (*Cfap45*$^{+/-}$) and homozygous (*Cfap45*$^{-/-}$) mutant sperm flagella by IFM. Samples are counterstained for ciliary axonemes (green) using anti-acetylated α tubulin (AcTub) antibody. Merge images include Hoechst stain to visualize nuclei (blue). White scale bars equal 10 µm. **c**,**d**, *n* = 12 images from 2 experiments. **e**, DNAH11 is not detectable in wild type mouse sperm lysates by IB. **f**, DNAH17 is detectable in wild type mouse sperm lysates by IB. Orange arrowhead indicates no detectable immunoreactive band within the range of the predicted molecular weight of murine DNAH11 (UniProt ID Q9QZH1, 516 kDa.). Green arrowhead indicates a detectable immunoreactive band within the range of the predicted molecular weight of murine DNAH17 (UniProt ID Q69Z23, 512 kDa). **e**,**f**, *n* = 3.

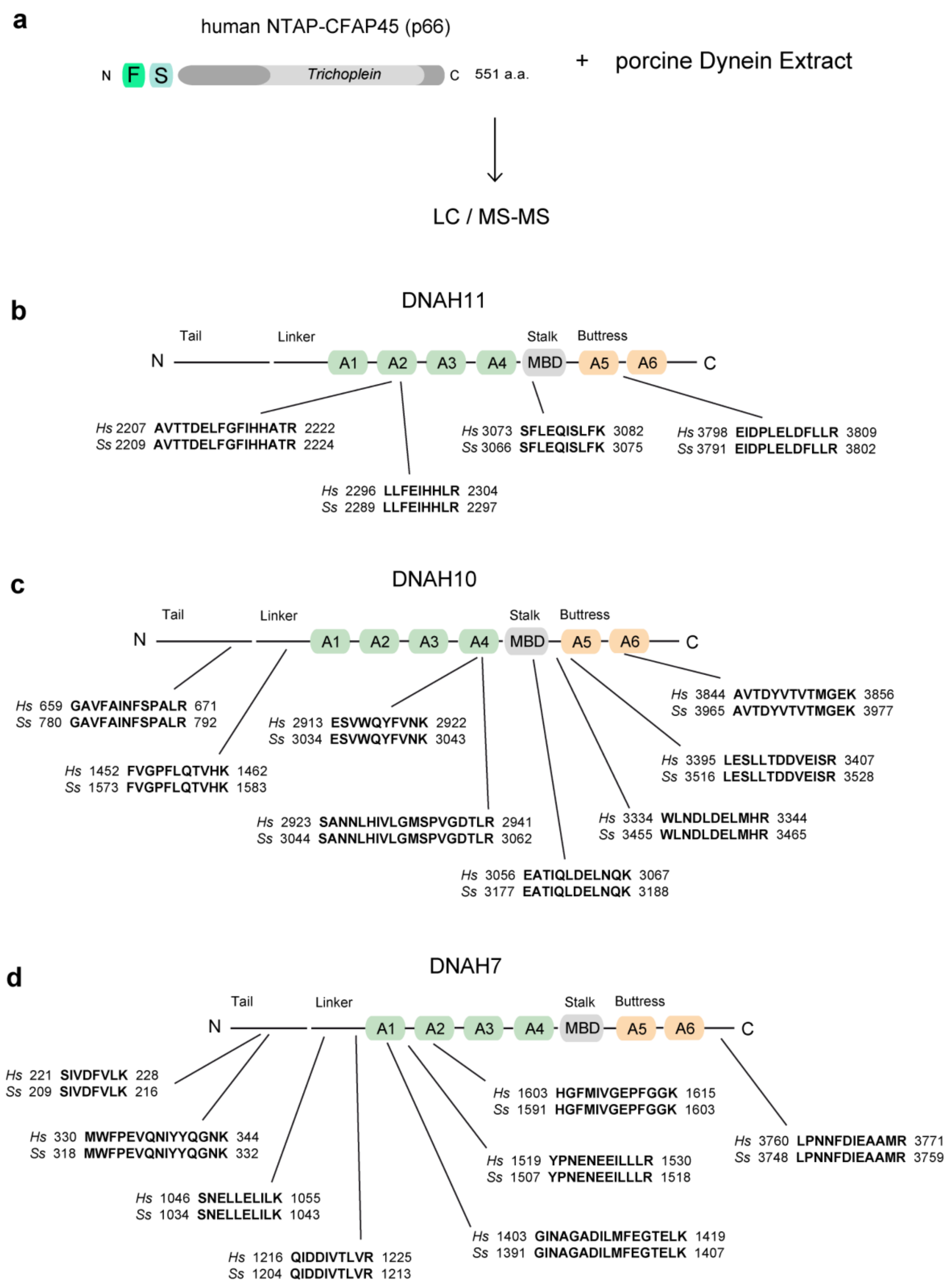

Supplementary Fig. 13 | Recombinant full length CFAP45 interacts with dynein ATPases including DNAH11. a, Tandem-affinity purified recombinant CFAP45 (as shown in **Fig. 7c**) is incubated with porcine dynein extracts to test whether full length recombinant CFAP45 asssociates with dynein ATPases. **b**, LC / MS-MS identifies four peptides specific for DNAH11. **c**, LC / MS-MS identifies eight peptides specific for DNAH10. **d**, LC / MS-MS identifies eight peptides specific for DNAH7. These peptides are not identified in NTAP control immunoprecipitates. All porcine (*Ss*) peptides show 100% identity to respective human (*Hs*) dynein ATPases. See also **Supplementary Data 1** and **Methods**.

a

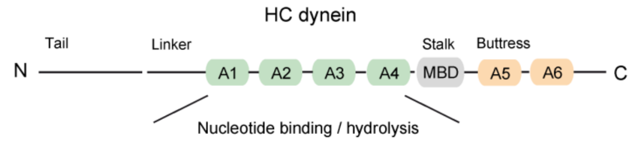

b

	A1	A2	A3	A4
DNAH1	ADP	ATP	ADP	ATP/ADP
DNAH2	ATP	ATP	ATP	ATP/ADP
DNAH3	ADP	ATP	ATP	ATP/ADP
DNAH5	ATP	ATP	ATP	ATP/ADP
DNAH6	ADP	ATP	ATP	ATP/ADP
DNAH7	ADP	ATP	ATP	ATP/ADP
DNAH8	ATP	ATP	ADP	ATP/ADP
DNAH9	ATP	ATP	ADP	ATP/ADP
DNAH10	ATP	ATP	ATP	ATP/ADP
DNAH11	ATP	ATP	ATP	ATP/ADP
DNAH12	ADP	ATP	ADP	X
DNAH14	ATP	ATP	X	X
DNAH17	ADP	ATP	ADP	ATP/ADP
DYNC1H1	ATP	ATP	ATP	ATP/ADP
DYNC2H1	ATP	ATP	ATP	ATP/ADP

c DNAH11

A1

	ATP	ADP
G	0.113	0.022
P	0.148	0.031
A	0.096	0.035
G	0.079	0.030
T	0.151	0.029
G	0.410	0.250
K	0.581	0.334
T	0.176	0.415
E	0.227	0.264
T	0.337	0.128

A2

	ATP	ADP
G	0.060	0.036
N	0.275	0.168
A	0.095	0.236
G	0.540	0.733
T	0.471	0.636
G	0.476	0.973
K	0.982	0.843
S	0.722	0.995
K	0.154	0.512

A3

	ATP	ADP
G	0.098	0.013
N	0.123	0.683
A	0.172	0.460
G	0.657	0.986
V	0.621	0.962
G	0.247	0.837
K	0.956	0.740
T	0.671	0.852
V	0.209	0.853

A4

	ATP	ADP
G	0.040	0.132
V	0.352	0.924
G	0.985	0.667
G	0.970	1.000
S	0.972	0.965
G	0.955	0.995
K	0.934	**0.963**
Q	0.344	0.523
S	0.930	0.933

d DNAH17

A1

	ATP	ADP
G	0.115	0.023
P	0.146	0.033
A	0.093	0.536
G	0.081	0.530
T	0.151	0.529
G	0.416	0.750
K	0.585	**0.833**
T	0.185	0.917
E	0.226	0.757
T	0.302	0.115

A2

	ATP	ADP
G	0.061	0.034
N	0.236	0.194
A	0.092	0.258
G	0.500	0.639
S	0.440	0.638
G	0.455	0.968
K	0.979	0.769
S	0.738	0.994
Q	0.163	0.531

A3

	ATP	ADP
G	0.135	0.006
N	0.056	0.242
A	0.084	0.134
G	0.185	0.139
T	0.184	0.390
G	0.123	0.305
K	0.255	**0.383**
S	0.500	0.510
V	0.067	0.299

A4

	ATP	ADP
G	0.044	0.129
V	0.343	0.914
G	0.984	0.631
G	0.975	1.000
S	0.972	0.967
G	0.953	0.997
K	0.939	**0.977**
Q	0.366	0.580
S	0.937	0.905

Supplementary Fig. 14 | ATP- and ADP-binding probability profiles of human dynein ATPase domains.a, Human dynein ATPases contain four centrally located ATPase (AAA1-AAA4, designated A1-A4) domains. **b**, *In silico* analysis (NSitePred) reveals variable nucleotide binding probability to ATP or ADP among human dynein ATPase domains. Consistent with its annotation (Uniprot), NSitePred did not identify probable nucleotide binding motifs in the A3 and A4 domains of DNAH14 (indicated by red 'X'). In contrast to its annotation (UniProt), NSitePred did not identify probable nucleotide binding motifs in the A4 domain of DNAH12. See also **Methods**. **c**, Similar to other dynein ATPases, the A4 domain of DNAH11 (with exception to DNAH12 and DNAH14) show high

binding probability to both ATP and ADP **d**, Similar to DNAH1, both the A1 and A3 domains as well as the A4 domain of DNAH17 show higher binding probability for ADP over ATP. For **c**,**d**, Gray highlights lysine (K) residue that is critical for nucleotide binding. This analysis suggests that among dynein ATPases, a) the AAA1 and AAA3 domains show variable probability for either ATP or ADP binding, b) the AAA2 domain shows an almost invariant probability for ATP binding, consistent with previous observations that AAA2 is likely constitutively bound to ATP throughout the hydrolysis cycle[11], and c) the AAA4 domain shows an almost invariant profile competitive for both ATP and ADP binding, suggesting that AAA4 may be particularly sensitive to fluctuations in the levels of ATP and ADP.

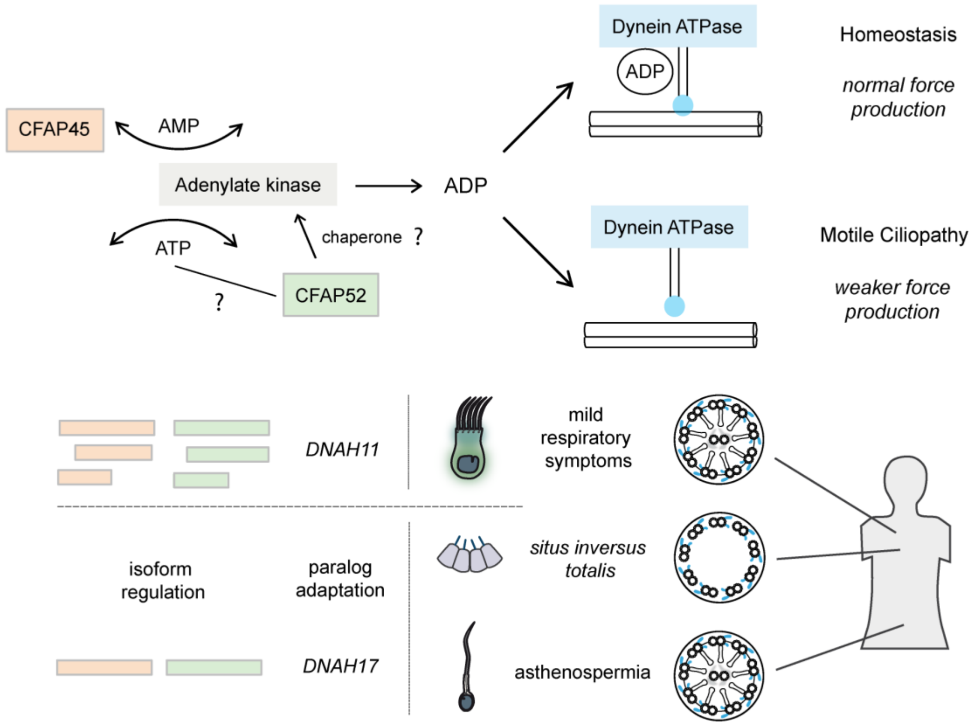

Supplementary Fig. 15 | CFAP45 deficiency disrupts adenine nucleotide homeostasis. Model whereby CFAP45 mediates adenine nucleotide homeostasis via AMP transfer to AK8, thereby favoring ADP production. Under conditions of ADP homeostasis, balanced levels of ADP target ADP-sensitive regulatory ATPase domains (e.g., AAA4) to ensure that dynein ATPases maintain normal force production through affinity for axonemal MTDs. Under conditions of CFAP45 deficiency, ADP-sensitive regulatory domains of dynein ATPases may bind ATP by default, short-circuiting the mechano-chemical cycle (ATP hydrolysis at AAA1) and also affecting affinity of dynein ATPases for axonemal MTDs. In this scenario, the source of ATP as a phosphate donor is not specified. CFAP52 contains highly conserved motifs for ATP binding (**Fig. 5p**) but its potential ATP binding function was not tested. CFAP52 may have other functions, e.g. chaperone, (see additional associations including chaperone family proteins in **Supplemental Data 1**) that account for its dual association with CFAP45 and AK8. Tissue-specific control of ADP homeostasis likely includes isoform regulation of AMP-binding CFAP45 and also paralog adaptation of dynein ATPases. We previously reported panaxonemal localization of Gfp-Dnah11 in LRO cilia[12]; CFAP45 is also panaxonemal in wild type LRO cilia (**Fig. 4d,e**). Compartmentalization of the respiratory axoneme into distinct proximal and distal regions may also help explain the mild respiratory phenotype of CFAP45 deficiency; this compartmentalization is not apparent in motile LRO monocilia and helps explain the penetrant phenotype of *situs inversus totalis* in both *DNAH11* and *CFAP45* deficiency. See also **Fig. 7** and **Methods**.

References

1. Lucas, J.S. et al. European Respiratory Society guidelines for the diagnosis of primary ciliary dyskinesia. *Eur. Respir. J*. **49**, 1601090 (2017).
2. Shapiro, A.J. et al. Diagnosis, monitoring, and treatment of primary ciliary dyskinesia: PCD foundation consensus recommendations based on state of the art review. *Pediatr. Pulmonol*. **51**, 115-32 (2016).
3. Ta-Shma, A. et al. A human laterality disorder associated with a homozygous WDR16 deletion. *Eur. J. Hum. Genet*. **23,** 1262-5 (2015).
4. Gherman, A., Davis, E.E. & Katsanis, N. The ciliary proteome database: an integrated community resource for the genetic and functional dissection of cilia. *Nat Genet*. **38**, 961-2 (2006).
5. Inglis, P.N., Boroevich, K.A. & Leroux, M.R. Piecing together a ciliome. *Trends Genet*. **22**, 491-500 (2006).
6. Blackburn, K., Bustamante-Marin, X., Yin, W., Goshe, M.B. & Ostrowski, L.E. Quantitative Proteomic Analysis of Human Airway Cilia Identifies Previously Uncharacterized Proteins of High Abundance. *J Proteome Res*. **16**, 1579-1592 (2017).
7. Adachi, J. et al. Proteome-wide discovery of unknown ATP-binding proteins and kinase inhibitor target proteins using an ATP probe. *J Proteome Res*. **13**, 5461-70 (2014).
8. Amaral, A. et al. Identification of proteins involved in human sperm motility using high-throughput differential proteomics. *J Proteome Res*. **13**, 5670-84 (2014).
9. Loges, N.T. et al. Recessive DNAH9 Loss-of-Function Mutations Cause Laterality Defects and Subtle Respiratory Ciliary-Beating Defects. *Am J Hum Genet*. **103**, 995-1008 (2018).
10. Hastie, A.T. et al. Isolation of cilia from porcine tracheal epithelium and extraction of dynein arms. *Cell Motil Cytoskeleton* **6**, 25-34 (1986).
11. Schmidt, H., Gleave, E.S. & Carter, A.P. Insights into dynein motor domain function from a 3.3-Å crystal structure. *Nat. Struct. Mol. Biol*. **19**, 492-7 (2012).
12. Dougherty, G.W. et al. DNAH11 Localization in the Proximal Region of Respiratory Cilia Defines Distinct Outer Dynein Arm Complexes. *Am. J. Respir. Cell Mol*. Biol. **55**, 213-24 (2016).
