# Supplementary material for: CFAP45 deficiency causes situs abnormalities and asthenospermia by disrupting an axonemal adenine nucleotide homeostasis module
Source: Nat Commun. 2020 Nov 2;11:5520. doi: 10.1038/s41467-020-19113-0 (PMC7606486; doi:10.1038/s41467-020-19113-0)
Supplement: Supplementary file 3 — Description of Additional Supplementary Files [file 41467_2020_19113_MOESM3_ESM.pdf]

Description of Additional Supplementary Files

File Name: Supplementary Data 1
Description: Overview of CFAP45 and CFAP52 associations

File Name: Supplementary Movie 1
Description: Flagellar beating of healthy control human sperm

File Name: Supplementary Movie 2
Description: Flagellar beating of *CFAP45*-deficient sperm from individual OP-28 II1

File Name: Supplementary Movie 3
Description: Nodal ciliary beating of a *Cfap45* $^{+/-}$ embryo

File Name: Supplementary Movie 4
Description: Nodal ciliary beating of a *Cfap45* $^{-/-}$ embryo

File Name: Supplementary Movie 5
Description: Flagellar beating of sperm from a *Cfap45* $^{+/-}$ male mouse

File Name: Supplementary Movie 6
Description: Flagellar beating of sperm from a *Cfap45* $^{-/-}$ male mouse
