# Supplementary material for: CFAP45 deficiency causes situs abnormalities and asthenospermia by disrupting an axonemal adenine nucleotide homeostasis module
Source: Nat Commun. 2020 Nov 2;11:5520. doi: 10.1038/s41467-020-19113-0 (PMC7606486; doi:10.1038/s41467-020-19113-0)
Supplement: Supplementary file 11 — Reporting Summary [file 41467_2020_19113_MOESM11_ESM.pdf]

nature research

Corresponding author(s): Heymut Omran, M.D.

Last updated by author(s): Jun 25, 2020

Reporting Summary

Nature Research wishes to improve the reproducibility of the work that we publish. This form provides structure for consistency and transparency in reporting. For further information on Nature Research policies, see our Editorial Policies and the Editorial Policy Checklist.

Statistics

For all statistical analyses, confirm that the following items are present in the figure legend, table legend, main text, or Methods section.

n/a	Confirmed	
☐	☒	The exact sample size (*n*) for each experimental group/condition, given as a discrete number and unit of measurement
☐	☒	A statement on whether measurements were taken from distinct samples or whether the same sample was measured repeatedly
☐	☒	The statistical test(s) used AND whether they are one- or two-sided<br>*Only common tests should be described solely by name; describe more complex techniques in the Methods section.*
☒	☐	A description of all covariates tested
☒	☐	A description of any assumptions or corrections, such as tests of normality and adjustment for multiple comparisons
☐	☒	A full description of the statistical parameters including central tendency (e.g. means) or other basic estimates (e.g. regression coefficient) AND variation (e.g. standard deviation) or associated estimates of uncertainty (e.g. confidence intervals)
☒	☐	For null hypothesis testing, the test statistic (e.g. *F*, *t*, *r*) with confidence intervals, effect sizes, degrees of freedom and *P* value noted<br>*Give P values as exact values whenever suitable.*
☒	☐	For Bayesian analysis, information on the choice of priors and Markov chain Monte Carlo settings
☒	☐	For hierarchical and complex designs, identification of the appropriate level for tests and full reporting of outcomes
☒	☐	Estimates of effect sizes (e.g. Cohen's *d*, Pearson's *r*), indicating how they were calculated

Our web collection on statistics for biologists contains articles on many of the points above.

Software and code

Policy information about availability of computer code

Data collection	We did not use unpublished custom algorithm or software to collect or analyze data in this study.
Data analysis	Publicly available databases were used to analyze data and are indicated by respective URLs in the Data availability statement (see Data below)

For manuscripts utilizing custom algorithms or software that are central to the research but not yet described in published literature, software must be made available to editors and reviewers. We strongly encourage code deposition in a community repository (e.g. GitHub). See the Nature Research guidelines for submitting code & software for further information.

Data

Policy information about availability of data

All manuscripts must include a data availability statement. This statement should provide the following information, where applicable:

- Accession codes, unique identifiers, or web links for publicly available datasets
- A list of figures that have associated raw data
- A description of any restrictions on data availability

We have included the following data availability statement in the manuscript:
The data that support the findings of this study are available from the corresponding author upon reasonable request. WES data is available in a restricted manner due to privacy considerations and is contingent on the approval of the Institutional Ethics Review Board from the University Hospital Münster (Germany) and ethics committees from collaborating institutions. Proteomic data are available via ProteomeXchange with identifier PXD019806. Data shown in Fig. 7b, Fig. 7d,e, and Supplementary Fig. 9a-c are provided as a Source Data File. The following publicly available databases were used:
gnomAD, http://gnomad.broadinstitute.org/;

1000 Genomes, http://browser.1000genomes.org/index.html; MGI, http://www.informatics.jax.org/;
GenBank, https://www.ncbi.nlm.nih.gov/genbank/; Uniprot, http://www.uniprot.org/;
Ensembl, http://www.ensembl.org/index.html; Pfam, http://pfam.xfam.org/;
Varbank, https://varbank.ccg.uni-koeln.de/; PROVEAN, http://provean.jcvi.org/index.php;
Polyphen-2, http://genetics.bwh.harvard.edu/pph2/;
DAVID Bioinformatics Resources 6.7, https://david-d.ncifcrf.gov/;
Ciliome Database, http://www.sfu.ca/~leroux/ciliome_home.htm.;
The Human Protein Atlas, http://www.proteinatlas.org/;
NsitePred, http://biomine.cs.vcu.edu/servers/NsitePred/

Field-specific reporting

Please select the one below that is the best fit for your research. If you are not sure, read the appropriate sections before making your selection.

- [x] Life sciences
- [ ] Behavioural & social sciences
- [ ] Ecological, evolutionary & environmental sciences

For a reference copy of the document with all sections, see nature.com/documents/nr-reporting-summary-flat.pdf

Life sciences study design

All studies must disclose on these points even when the disclosure is negative.

Sample size	Sample size for experiments was not predetermined and dependent on availability and general guideline to achieve at least two independent experiments. For example, adenine nucleotide rescue experiments with transgenic mice could generally achieve n > 5 independent experiments (Fig. 7), whereas sperm experiments in human individuals (Supp. Fig. 2) (n = 1, 2 independent measurements from same sample) was limited to patient availability.
Data exclusions	No data were excluded from the analysis.
Replication	The number of replicates and independent experiments is provided in the main text and figure legends for all experiments. The experiments yielded similar results that supported a trend; the overall conclusion was based on trends collated from results using various complementary techniques.
Randomization	Randomization was not applied to individuals as they were selected for a specific disease phenotype; likewise, randomization of heterozygous and homozygous mice is not applicable because they both received no or the same treatment; Schmidtea morphants were analyzed randomly from a pool of either control or targeted RNA.
Blinding	Immunoflurescence analysis of samples was blinded and replicated at least two independent times; blinding is not feasible for Schmidtea morphant analysis as it requires injection at multiple timepoints; blinded analysis of nodal ciliary beating was performed before genotype analysis; blinded analysis for other mouse experiments is not feasible because genotyping of transgenic mice was known before experimental analysis.

Reporting for specific materials, systems and methods

We require information from authors about some types of materials, experimental systems and methods used in many studies. Here, indicate whether each material, system or method listed is relevant to your study. If you are not sure if a list item applies to your research, read the appropriate section before selecting a response.

Materials & experimental systems

n/a	Involved in the study	
[ ]	[x]	Antibodies
[ ]	[x]	Eukaryotic cell lines
[x]	[ ]	Palaeontology and archaeology
[ ]	[x]	Animals and other organisms
[ ]	[x]	Human research participants
[x]	[ ]	Clinical data
[x]	[ ]	Dual use research of concern

Methods

n/a	Involved in the study	
[x]	[ ]	ChIP-seq
[x]	[ ]	Flow cytometry
[x]	[ ]	MRI-based neuroimaging

Antibodies

Antibodies used	The following rabbit polyclonal antibodies were purchased from Atlas Antibodies: anti-CFAP45, HPA043618; anti-DNAH11, HPA045880; anti-CFAP52, HPA023247; anti-IQCD, HPA048782; anti-DNAI1, HPA021649; anti-DNAH9, HPA052641; anti-DNAH5, HPA037470; anti-AK8, HPA021445; anti-DNAH17, HPA024354. Polyclonal anti-DNAH5 (amino acids 42-325) and monoclonal anti-DNALI1 antibodies were reported (see Methods and References). Mouse monoclonal anti-acetylated α tubulin (T7451) and Hoechst

	33342 (B2261) were purchased from Sigma Aldrich. Mouse monoclonal anti-DNAH11 (non-commercial, undiluted hybridoma supernatant) was described (see Methods and References). The following secondary antibodies were purchased from Dianova for IFM and used at a 1:200 dilution: anti-rabbit Rhodamine Red-X (711-295-152), and anti-mouse Cy3 (715-166-150). Anti-mouse Alexa Fluor 488 (A11029), anti-mouse Alexa Fluor 546 (A11030), anti-rabbit Alexa Fluor 488 (A11034) and anti-rabbit Alexa Fluor 546 (A11035) were purchased from Invitrogen and used at a 1:200 and 1:1,000 dilution for human and mouse samples, respectively. Appropriate controls including secondary antibodies alone were performed. Normal rabbit (sc-2027) IgG (Santa Cruz Biotechnology Incorportated) was used as control for immunoprecipitations. HRP-linked anti-rabbit (NA934V) and anti-mouse (NA931V) antibodies (GE Healthcare) were used for immunoblotting. Please see Methods for antibody dilutions relating to specific experiments.
Validation	To our knowledge, this study provides the first extensive validation of human anti-CFAP45 (Atlas antibodies, HPA043618) and human anti-CFAP52 (Atlas antibodies, HPA023247) based on testing genetically-null samples in human and mouse by immunofluorescence microscopy, immunoprecipitation and immunoblotting of recombinant human CFAP45 and CFAP52, native immunoprecipitation and mass spectrometry of CFAP45 and CFAP52 from pig respiratory cells, and immunofluorescence and immunoblotting of native CFAP45 and CFAP52 in tissues with motile cilia and flagella in human, mouse, and pig.

Eukaryotic cell lines

Policy information about cell lines

Cell line source(s)	HEK293 cell line CRL-1573 was obtained by ATCC.
Authentication	HEK293 cell line CRL-1573 obtained by ATCC was not further authenticated.
Mycoplasma contamination	The HEK293 cell line used in this study tested negative for Mycoplasma contamination.
Commonly misidentified lines (See ICLAC register)	No commonly misidentified cell lines were used in this study.

Animals and other organisms

Policy information about studies involving animals; ARRIVE guidelines recommended for reporting animal research

Laboratory animals	A clonal line of asexual S. mediterranea (BCN-10, originally provided by E. Saló) was maintained at 20°C in Montjuïch salts solution. C57BL/6 mice were used to generate a Cfap45-/- transgenic line by CRISPR-Cas9. Mice were housed under specific-pathogen-free (SPF) conditions in cages (according to EU-guideline 2010/63/EU) with bedding and nesting material containing up to five animals and monitored by qualified members of the working group. The housing temperature was 22-24°C constantly and a day and night cycle (12:12 hours) was maintained. Water and food were available ad libidum. Approximately equal numbers of males and females ranging from embryonic day 7.5 to postnatal day 60 were examined. Please see Methods for additional information.
Wild animals	This study did not use samples obtained from wild animals. Domesticated pig (Sus scrofa) was used for pig respiratory samples and obtained by Holstiege Farm (Roxel, Germany).
Field-collected samples	This study did not involve samples collected from the field.
Ethics oversight	All mouse experiments were approved by local government authorities (AZ84-02.05.20.12.163; 84-02.05.20.12.164, Landesamt für Natur, Umwelt und Verbraucherschutz Nordrhein-Westfalen, University Hospital Muenster; FBS-12-019, Osaka University; AH28-01, RIKEN Center for Developmental Biology) and complied with ethical regulations.

Note that full information on the approval of the study protocol must also be provided in the manuscript.

Human research participants

Policy information about studies involving human research participants

Population characteristics	Individuals suspected to have a motile ciliopathy participated in PCD-study protocols that were approved by the Institutional Ethics Review Board from the University Hospital of Muenster (Germany) and collaborating institutions. Inclusion criteria were chronic upper respiratory symptoms (e.g., rhinosinusitis, otitis media) and LRA abnormalities (e.g., situs inversus totalis) with or without asthenospermia / male infertility. The diagnosis of PCD is supported by evidence of destructive lower airway disease (bronchiectasis), hallmark features of dyskinetic or immotile respiratory cilia by high-speed videomicroscopy analysis (HVMA), low nasal nitric oxide (nNO) levels and structural defects of respiratory cilia by conventional transmission electron microscopy analysis (TEM).
Recruitment	Individuals without symptoms of chronic destructive airway disease as well as HVMA and nNO levels within normal range do not fulfill diagnostic criteria for PCD and were recruited as participants for the motile ciliopathy cohort described in this study through the University Hospital Muenster (Muenster, Germany), Hadassah-Hebrew University Medical Center (Jerusalem, Israel), and collaborating institutions.
Ethics oversight	Individuals suspected to have a motile ciliopathy participated in PCD-study protocols that were approved by the Institutional Ethics Review Board from the University Hospital of Muenster (Germany) and collaborating institutions. Individuals provided signed and informed consent to participate in this study. Human sperm analyses of individual OP-28 II1 and healthy donors were performed in accordance with the standards set by the Declaration of Helsinki. Samples of human semen were obtained from healthy donors and individual OP-28 II1 with their prior written consent, under approval of the institutional ethical committees of the medical association Westfalen-Lippe and the medical faculty of the University of Münster (4INie).

Note that full information on the approval of the study protocol must also be provided in the manuscript.
